# A case report of medically managed esophageal fistula due to complicated esophageal tuberculosis

**DOI:** 10.1016/j.ijscr.2022.106883

**Published:** 2022-02-25

**Authors:** Najib Mohamed Salad, Ismail A. Ali, Yahye Garad Mohamed

**Affiliations:** a Department of General Surgery, Mogadishu Somali Turkey, Recep Tayyip Erdogan Training and Research Hospital, Mogadishu, Somalia; b Department of Radiology, Mogadishu Somali Turkey, Recep Tayyip Erdogan Training and Research Hospital, Mogadishu, Somalia

**Keywords:** Esophageal tuberculosis, Esophageal fistula, Case report

## Abstract

**Introduction and importance:**

Esophageal TB is a relatively uncommon condition. Mostly, the esophagus can be affected by tuberculosis through direct spread or from mediastinal nodes (rarely from the lungs or bloodstream). The most common symptom is dysphagia, and the diagnosis is confirmed by histology. If left untreated, esophageal tuberculosis can result in bleeding, perforation, fistula formation, aspiration pneumonia, lethal hematemesis, traction diverticula, and esophageal strictures.

**Case presentation:**

This is a rare case report of an esophageal fistula caused by tuberculosis in a patient presenting with a cough on eating and weight loss. The patient was subjected to upper gastrointestinal endoscopy, which revealed a cervical esophagus fistula 20 cm from the upper central incisors. Histopathology revealed inflammatory lesions with epithelioid granulomas (granulomatous disease). A mycobacterium sputum examination was performed; the smear was negative. The patient was managed conservatively with anti-tuberculosis treatment (ATT). A follow-up endoscopy after two months revealed that the fistula was closed and clinically improved.

**Clinical discussion:**

The quick clearance of contaminated sputum by coordinated peristalsis, paired with upright posture and an intact lower esophageal sphincter, limits the organism's exposure to the esophagus.

**Conclusion:**

Despite the disease's rarity, if not delayed, it can be efficiently managed with ATT to avoid major complications like esophageal perforation, which necessitates surgery.

## Highlights

• Esophageal fistula due to complicated esophageal tuberculosis can be efficiently managed with ATT to avoid major complications like esophageal perforation, which necessitates surgery.
• This case highlights non-surgical management of the esophageal fistula due to complicated esophageal tuberculosis.
• Esophageal fistula is one of the rare clinical manifestations of esophageal tuberculosis, and the most common symptom is dysphagia.

## 1 Introduction

A global estimate of 10.0 million people fell ill with TB in 2019, a number that has been decreasing gradually in recent years [1]. Southeast Asia and Africa accounted for nearly 70% of total global TB, according to the WHO [2].

Somalia has one of the highest incidence rates of TB in the world. Every year, approximately 12,000 people test positive for sputum.

Tuberculosis is an airborne infectious disease caused by organisms of the mycobacterium tuberculosis complex. Although primarily a pulmonary pathogen (pulmonary tuberculosis), tuberculosis can cause disease in almost any part of the body (extra-pulmonary tuberculosis).

Esophageal fistula is one of the rare clinical manifestations of esophageal tuberculosis.

Esophageal tuberculosis has two types: primary and secondary. The secondary one is more common as primary involvement is extremely rare due to the intrinsic protection of the esophagus.

## 2 Case presentation

A 23-year-old Somali male complains of seven months of productive cough and pleuritic chest pain associated with some episodes of intermittent fever.

After several months of these symptoms, he also developed a cough on eating and halitosis. The patient-reported mild weight loss over that moment.

Vital signs were stable, and a physical examination of the abdomen and other systems was normal. The laboratory investigations included the following: Hemoglobin 12 (normal 13–17 g/dL); white blood cell 9.01 (normal 4–10 10^6^/L) (segmented neutrophils 68.2%, lymphocytes 16.5%); erythrocyte sedimentation rate 108 (normal 20 mm/h); C-reactive protein 69.3 (normal 0–10 mg/L); albumin 4.2 (normal 3.5–5 g/dL); aspartate aminotransferase 16 (normal).

An initial chest X-ray revealed a right lung apex where there is local infiltration.

**Fig. 1:**
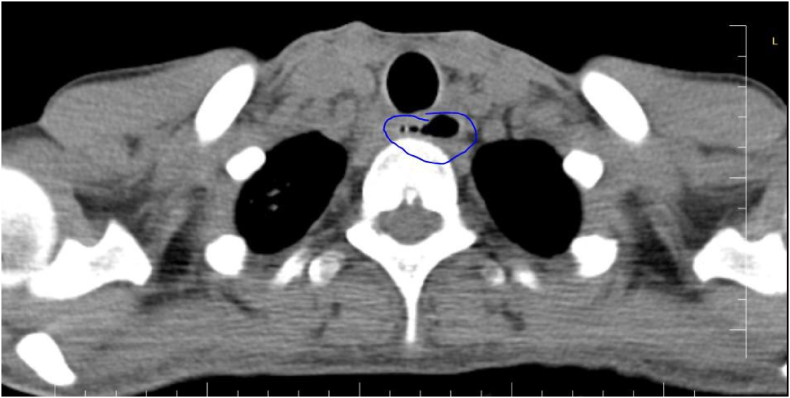
Axial chest CT shows fistulate tract, which contains air and communicates with the esophagus.

Further workup was obtained with a CT chest, which showed in the right lung apex there was local peribronchial infiltration and at the proximal esophagus level, there was a 2 cm hypodense lesion with air content that communicated with the esophagus (Fig. 1). To determine the etiology of this sinus, the patient underwent esophagogastroduodenoscopy evaluation, which revealed a cervical esophagus fistula 20 cm from the upper central incisors, and a biopsy was taken.

After that, he was admitted for medical observation. The patient was kept NPO for one month after an NG tube was inserted. Several days later, the histological examination of the biopsy showed inflammatory lesions with epithelioid granulomas (granulomatous disease) and a mycobacterium sputum examination was performed; the smear was negative. The human immunodeficiency virus (HIV) serology was negative. Our patient started empirical antituberculosis therapy with iso-isoniazid, rifampicin, pyrazinamide, and ethambutol with symptomatic improvement. After two months, we repeated the endoscopy, and the sinus tract is closed. The patient has clinically improved and is also under follow-up.

## 3 Discussion and conclusion

Esophageal TB is a relatively uncommon condition. The most common cause is direct spread from mediastinal nodes (rarely from the lungs or bloodstream). The most common symptom is dysphagia, and the diagnosis is confirmed by histology. If left untreated, esophageal tuberculosis can result in bleeding, perforation, fistula formation, aspiration pneumonia, lethal hematemesis, traction diverticula, and esophageal strictures.

Extrapulmonary tuberculosis (EPTB) is when the tuberculous mycobacterium invades areas outside the pulmonary parenchyma and has nonspecific clinical findings that develop insidiously, mimicking other noninfectious conditions [3], [4]. Esophageal Tb is the least common site of Tb in the GI tract [5].

In autopsy studies, esophageal involvement was found in only 0.15% of patients who died of tuberculosis [6].

.Primary esophageal tuberculosis is defined as esophageal involvement with no other systemic manifestation of tuberculosis. Primary esophageal tuberculosis is rare because the quick clearance of contaminated sputum by coordinated peristalsis, paired with upright posture and an intact lower esophageal sphincter, limits the organism's exposure to the esophagus [7]. The symptoms of esophageal tuberculosis include dysphagia, odynophagia, chest pain, low-grade fever, and weight loss [7], [8]. In our case, the patient had been experiencing seven months of productive cough and pleuritic chest pain associated with some episodes of intermittent fever and also cough on eating and halitosis. He likely experienced complications of esophageal TB because his symptoms were not timely addressed due to his delay in seeking medical treatment. Most patients, however, with esophageal involvement of TB will also have chest imaging findings consistent with pulmonary TB. In our case, chest imaging showed in the right lung apex that there was local peribronchial infiltration. The solitary ulcer with an excavated base and raised edges is the most common endoscopic finding of esophageal TB case [9]. Esophagogastroduodenoscopy evaluation revealed a cervical esophagus fistula 20 cm from the upper central incisors, and a biopsy was taken.

Individuals with esophageal tuberculosis involvement may be effectively treated using RIPE therapy for a duration of six to nine months [10], [11]. In our case, we started RIPE therapy after the biopsy result and, several weeks later, the patient clinically improved and now he is taking TB treatment.

This work has been reported in line with the SCARE 2020 criteria [12].

## Abbreviations

TB: Tuberculosis
ATT: Anti-tuberculosis treatment
RIPE: Rifampin, isoniazid, pyrazinamide, and ethambutol (treatment for TB)
NPO: Nothing by mouth
NG tube: Nasogastric tube

## Consent

Written informed consent was obtained from the patient for the publication of this case report and accompanying images. A copy of the written consent is available for review by the Editor-in-Chief of this journal on request.

## Provenance and peer review

Not commissioned, externally peer-reviewed.

## Ethical approval

Ethical approval was waived by the ethical committee of Mogadishu Somali Turkey, Recep Tayyip Erdogan Training and Research Hospital.

## Funding

No funding was received.

## Guarantor

Najib Mohamed Salad.

## Research registration number

N/a.

## CRediT authorship contribution statement

Najib Mohamed Salad: Conceptualization, Data curation, Visualization, Investigation Writing, Original draft preparation
Ismail A. Ali: Supervision, Validation
Yahye Garad: Writing, Reviewing, and Editing.

## Declaration of competing interest

o This manuscript has not been submitted to, nor is it under review at, another journal or other publishing venue.
o The authors have no affiliation with any organization with a direct or indirect financial interest in the subject matter discussed in the manuscript
